# The complete chloroplast genome and phylogenetic analysis of *Begonia pulchrifolia*, a near endangered Begoniaceae plant

**DOI:** 10.1080/23802359.2019.1660268

**Published:** 2019-09-02

**Authors:** Jing Fan, Xiao-Jie Li, Ce-Hong Li, Bai-Nan Yang

**Affiliations:** a College of Life Sciences, Leshan Normal University, Leshan, China;; b Emei Mountain Biotic Resource Experimental Station, Sichuan Provincial Institute of Natural Resource Science, Emeishan, China

**Keywords:** *Begonia pulchrifolia*, complete chloroplast genome, Begoniaceae, Illumina sequencing

## Abstract

*Begonia pulchrifolia* is a perennial herb of the genus Begonia and is a near endangered species. Traditional morphological delimitation of *B. pulchrifolia* is sometimes confused with similar species; therefore, further systematic classification is urgently needed. To enrich the genetic information and guide its molecular identification, the complete chloroplast genome of *B. pulchrifolia* was sequenced and illustrated. The complete chloroplast genome has a total length of 169,589 bp, containing a large single-copy (LSC, 76,057 bp) and a small single-copy region (SSC, 18,320 bp) separated by a pair of inverted repeat (IR_S_, 37,606 bp) regions. The overall GC content is 35.56%. A total of 142 genes were annotated, including 91 protein coding genes, 8 rRNA genes and 43 tRNA genes. Phylogenetic analysis showed that *B. pulchrifolia* is more closely related to *Momordica charantia* and *Gomphogyne cissiformis*. This study enriches the genetic information of *B. pulchrifolia* and contributes to its accurate molecular identification.

The genus *Begonia* is one of the most diverse species in the world, with more than 1800 species of plants, and is considered the sixth largest genus of angiosperms (Tian et al. 2018). It was widely used as a garden plant for its elegant shape and beautiful foliage (Kiyokawa et al. 1996). Interestingly, some species of *Begonia* were also used as food and medicine (Basurto-Peña et al. 2003; Guan et al. 2007). The number of *Begonia* in China is expected to be 250 to 300 species. However, as a result of excessive collection and habitat destruction, some wild *Begonias* have now become rare or even extinct (Tian et al. 2018). In November 2013, Dai-Ke Tian, a famous expert in the classification of *Begonia*, found a potential new species of *Begonia* in the Herbarium of the Health Science Center, Peking University, which was collected 30 years ago from Mount Emei, China. He then identified the species using the internal transcribed spacer (ITS) of rDNA and named the new species *Begonia pulchrifolia* D.K. Tian & C.H. Li (Tian et al. 2015). Compared with the single gene identification of species, the chloroplast genome has more genetic information and has been widely used in molecular identification (Fan et al. 2019). However, the chloroplast genome of *B. pulchrifolia* has not been reported. Thus, the chloroplast genome sequence of *B. pulchrifolia* was detected and reported in this paper.

The leaves of *B. pulchrifolia* were sampled from Mount Emei, Sichuan Province, China (103°22′45″E, 29°35′42″N). The specimen (number: EM0517) was stored in the herbarium of Leshan normal university. Total genomic DNA was extracted by SDS method, genome sequencing was performed using Illumina HiSeq Xten platform, the collected raw sequences were quality controlled using NGSQC Toolkit v2.3.3 (Patel and Jain 2012), then assembled by SPAdes v. 3.11.0 (Bankevich et al. 2012), and finally annotated by Plann software (Huang and Cronk 2015). The complete chloroplast genome size of *B. pulchrifolia* is 169,589 bp (GenBank accession no. MN076318), consisting of two inverted repeat sequences (IRs, 37,606 bp each) separated by one large single-copy (LSC, 76,057 bp) and one small single-copy region (SSC, 18,320 bp). It contains 142 genes (114 unique genes), including 91 protein-coding genes (79 unique genes), 8 rRNAs (4 unique genes) and 43 tRNA genes (31 unique genes). The total GC content of chloroplast genome is 35.56%.

To analyze the phylogeny of *B. pulchrifolia*, 34 complete chloroplast genomes were analyzed with MAFFT7.037 software (Katoh and Standley 2013), and then the Maximum Likelihood (ML) phylogenetic tree was constructed by Mega-X v10.0.5 software (Kumar et al. 2018). The parameters were set to as follows: 1000 bootstrap repetitions, General-time-reversible nucleotide substitution model incorporating a gamma distribution and invariant sites (GTR + G+I), complete deletion of gaps/missing data. The result of ML phylogenetic tree showed that *B. pulchrifolia* is closely related to *Momordica charantia* and *Gomphogyne cissiformis* (Figure 1). Our results provide useful resources for the species delimitation and evolutionary study of *B. pulchrifolia*.

**Figure 1.**
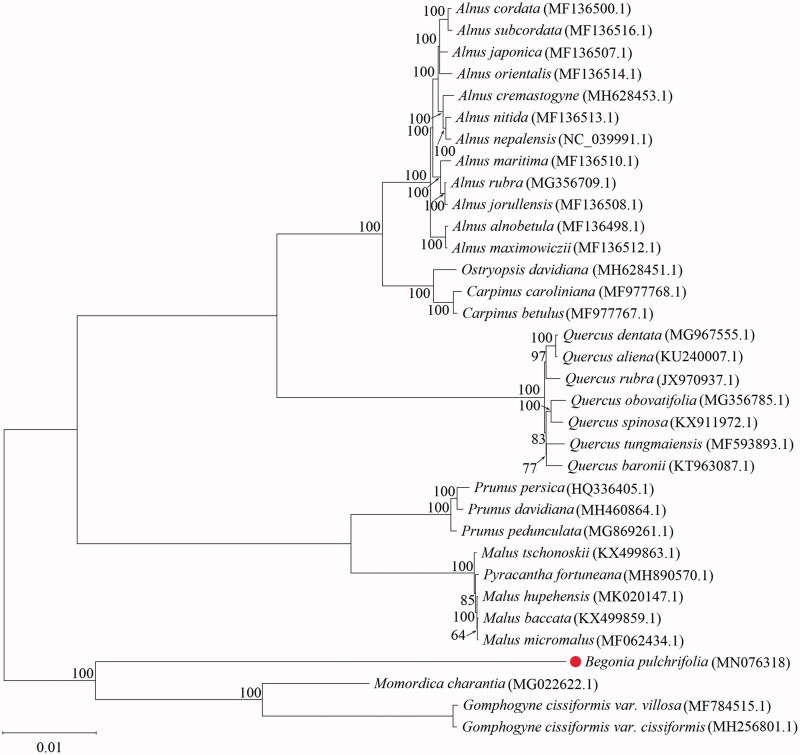
The maximum-likelihood (ML) phylogenetic tree was constructed from 34 complete plastome sequences using MEGA X software. *Note:* The number near each node represents the probability given by 1000 bootstrap analysis.
